# Exploration of *Trichoderma reesei* as an alternative host for erythritol production

**DOI:** 10.1186/s13068-024-02537-x

**Published:** 2024-06-27

**Authors:** Audrey Masi, Georg Stark, Johanna Pfnier, Robert L. Mach, Astrid R. Mach-Aigner

**Affiliations:** 1 Christian Doppler Laboratory for Optimized Expression of Carbohydrate-Active Enzymes, Institute of Chemical, Environmental and Bioscience Engineering, TU Wien, Gumpendorfer Str. 1a, 1060 Vienna, Austria; 2 Research Unit of Biochemical Technology, Institute of Chemical, Environmental and Bioscience Engineering, TU Wien, Gumpendorfer Str. 1a, 1060 Vienna, Austria

**Keywords:** Erythritol, *Trichoderma reesei*, Polyols, Design of experiments

## Abstract

**Background:**

Erythritol, a natural polyol, is a low-calorie sweetener synthesized by a number of microorganisms, such as *Moniliella pollinis*. Yet, a widespread use of erythritol is limited by high production costs due to the need for cultivation on glucose-rich substrates. This study explores the potential of using *Trichoderma reesei* as an alternative host for erythritol production, as this saprotrophic fungus can be cultivated on lignocellulosic biomass residues. The objective of this study was to evaluate whether such an alternative host would lead to a more sustainable and economically viable production of erythritol by identifying suitable carbon sources for erythritol biosynthesis, the main parameters influencing erythritol biosynthesis and evaluating the feasibility of scaling up the defined process.

**Results:**

Our investigation revealed that *T. reesei* can synthesize erythritol from glucose but not from other carbon sources like xylose and lactose. *T. reesei* is able to consume erythritol, but it does not in the presence of glucose. Among nitrogen sources, urea and yeast extract were more effective than ammonium and nitrate. A significant impact on erythritol synthesis was observed with variations in pH and temperature. Despite successful shake flask experiments, the transition to bioreactors faced challenges, indicating a need for further scale-up optimization.

**Conclusions:**

While *T. reesei* shows potential for erythritol production, reaching a maximum concentration of 1 g/L over an extended period, its productivity could be improved by optimizing the parameters that affect erythritol production. In any case, this research contributes valuable insights into the polyol metabolism of *T. reesei*, offering potential implications for future research on glycerol or mannitol production. Moreover, it suggests a potential metabolic association between erythritol production and glycolysis over the pentose phosphate pathway.

**Supplementary Information:**

The online version contains supplementary material available at 10.1186/s13068-024-02537-x.

## Novelty of the article

- Polyols are not a preferential carbon source for *T. reesei*
- The nitrogen source influences polyol biosynthesis
- Erythritol cannot be produced from every carbon source in *T. reesei*

## Introduction

Polyols, or sugar alcohols, are versatile compounds with applications spanning the food, pharmaceuticals, cosmetics, and plastic manufacturing sectors [1–3]. Defined by their molecular structure featuring multiple hydroxyl (–OH) groups, polyols include sorbitol, xylitol, glycerol, mannitol, and erythritol, each with distinct properties and applications [1, 4, 5]. Synthesized by a variety of bacteria, fungi, and plants, polyols naturally occur in some fruits, fermented foods, and plants [1, 6]. Among the polyol family, erythritol stands out due to its superior digestive tolerance and minimal impact on blood sugar levels, positioning it as an ideal sugar substitute. The growing demand for healthier, reduced-calorie food options underscores the market need for erythritol despite its limited production, which is attributed to high manufacturing costs. Erythritol is the sole polyol produced exclusively through biological processes [7]. Critical cost determinants in biotechnological processes are yield, choice of feedstock, and downstream requirements. Current erythritol production relies on the fermentation of a highly concentrated glucose solution by osmophilic yeast-like fungi, particularly *Moniliella pollinis, Aureobasidium* sp., *Torula* sp., and *Monilella megachiliensis* [4, 5, 8, 9]. The dependence on the mentioned glucose solutions, which are usually obtained from potatoes or corn starch hydrolysis, results in a costly process and sustainability challenges [10]. Identifying substrates beyond glucose hydrolyzed from starch for erythritol production is essential for reducing erythritol cost. While certain yeast strains have been engineered to enhance polyols yields or to utilize alternative carbon sources, the acceptance by consumers and the regulations related to genetically modified micro-organisms are an obstacle for offering such erythritol in specific markets, such as the European Union [5, 8, 9, 11–13].

In this context, microorganisms that are able to utilize lignocellulosic residues may provide a solution [10]. Lignocellulosic residues comprise agricultural or forestry waste and side products. Lignocellulose is the most abundant natural feedstock globally and is primarily composed of cellulose and hemicellulose, with hemicellulose composition varying based on the lignocellulosic material [14, 15]. Certain microorganisms can hydrolyze lignocellulose into cellulose and hemicellulose, further breaking down these polycarbohydrates into monomeric sugars [10, 14–16]. Cellulose predominantly releases glucose, while hemicellulose releases a mixture of xylose, arabinose, glucose, mannose, and galactose [14, 15, 17].

*Trichoderma reesei* is a saprotrophic fungus and, consequently, an example of such an organism. It is used for biotechnological production of extracellular cellulase and hemicellulase [16, 18, 19]. This native property of *T. reesei* positions it as a powerful candidate for harnessing lignocellulosic biomass, thereby addressing challenges related to feedstock availability, aligning with the principles of circular economy and sustainability, and contributing to cost reduction [19–21]. Another key attribute reinforcing *T. reesei* as a candidate for biotechnological usage is its Generally Recognized As Safe (GRAS) status conferred by the FDA [22, 23]. This status attests to the safety of *T. reesei* in producing compounds for pharmaceutical use and food.

The investigation of the potential of *T. reesei* for polyol production, specifically erythritol, remains limited [21, 24, 25]. Although *T. reesei* has been reported to synthesize small amounts of erythritol, an exploration of the parameters impacting erythritol production is lacking [21]. The present study followed this inquiry. We conducted preliminary tests to assess erythritol consumption by *T. reesei* and investigated whether an increase in glucose concentration, as observed in yeasts, would stimulate erythritol biosynthesis and prevent the eventual consumption of erythritol. Then, we investigated the influence of carbon and nitrogen sources on erythritol biosynthesis. This involved testing three distinct carbon sources and four nitrogen sources, and we optimized the best combination. Starting from the optimized carbon/nitrogen composition, we evaluated the possibility of using this process in bioreactors. The Design of experiment (DoE) method was used to investigate the relationship between carbon and nitrogen sources and, between pH and temperature. Altogether, this study provides valuable insights into polyol biosynthesis by *T. reesei*, with an emphasis on erythritol production.

## Materials and methods

### Strain and cultivation medium

The strain used is *T. reesei* QM6a∆tmus53, which is deficient in the non-homologous end-joining repair system and can be considered wild-type like as this modification does not impact polyol metabolism. The strain was maintained on malt extract (MEX) agar plates and was incubated at 30 °C for 3 to 4 days without light, followed by 2 to 3 days at room temperature under natural light to generate spores. MEX medium was prepared by mixing 30 g/L malt extract (Merck), 1 g/L peptone (Merck), and 15 g/L biological agar with tap water and sterilizing it by autoclaving.

For cultivation in shake flasks, the final concentration of the medium was 1 g/L MgSO_4_.7H_2_O, 4 g/L KH_2_PO_4_, 0.5 g/L NaCl, 0.1 g/L peptone, 0.5 g/L Tween 80, 5 mg/L FeSO_4_.7H_2_O, 17 mg/L MnSO_4_.H_2_O, 14 mg/L ZnSO_4_.7H_2_O, and 2 mg/L CaCl_2_.2H_2_O. The medium composition was adapted from previous protocols [21, 26]. Carbon and nitrogen source solutions were prepared separately, and the required solutions were mixed with the medium and sterile tap water before use. Glucose and glycerol solutions were prepared with ultrapure water and autoclaved. Xylose, lactose, erythritol, and nitrogen source solutions were prepared with ultrapure water and sterile-filtered. pH of urea solutions was adjusted to 4 with 1 M HCl. The components of the medium were separated into different stock solutions and mixed before use with sterile tap water, and the final pH was adjusted to 5 if necessary. The ten times concentrated solution containing MgSO_4_.7H_2_O, KH_2_PO_4_, NaCl, peptone, and Tween 80 and the 100-times concentrated solution containing FeSO_4_.7H_2_O, MnSO_4_.H_2_O, ZnSO_4_.7H_2_O, and CaCl_2_.2H_2_O were sterilized by autoclaving. Ultrapure water was prepared with an Arium® mini system (Sartorius). Sterile filtration of solutions was performed with Steritop® 0.2 μm PES, 500 mL (MilliporeSigma®), or 0.2 μm syringe filters VacuCap depending on the volume to filter.

### Spore suspension and inoculation

Spore suspension was prepared on the day of use. Spores scratched from the surface of a MEX plate were resuspended and vortexed in a sterile solution containing 0.8% NaCl and 0.05% Tween 80. The optical density of the spore solution was measured at 700 nm. Flasks were individually inoculated with spore suspension to an optical density of 0.05 in the final volume. Cultivations were performed in a volume of 100 mL medium in a 250-mL bottleneck shake flask incubated at 30 °C and stirred at 180 rpm. Cultivations were started with a pH of 5 unless stated otherwise in the results section, and pH was adjusted with 1 M HCl or 1 M NaOH.

### Cultivation in bioreactors (adapted from [21, 26])

Cultivation in bioreactors was performed in 2-L glass double-envelop bench bioreactors (Bioengineering) equipped with a Pt100 temperature probe, a pH probe, a sparger for aeration, an inlet for feed and pH regulation with HCl, and a sampling port. The cultivation medium described above was used without peptone and Tween 80. These two compounds are usually added to favor spore germination and avoid spore amalgamation. As bioreactors were inoculated with a pre-culture and not with spores, thus these compounds were not needed. The medium, tracer solution, and water volumes were pre-mixed and autoclaved in the bioreactor. A 400 g/L glucose stock solution was prepared in ultrapure water and autoclaved, and a 4 M urea solution was sterile-filtered.

After autoclaving the bioreactor, a 100 mL bottle containing 1 M HCl was connected to the reactor under sterile conditions. Inoculation was performed with a mycelium preculture, prepared in shake flasks—as described in the "Spore suspension and inoculation" paragraph—and incubated for 18 h. The preculture volume was 10% (v/v) of the final bioreactor volume and was injected sterilely through the sampling port of the bioreactor using a syringe.

For batch cultivations, the starting volume varied from 0.5 to 1.25 L. Urea and glucose solutions were added to the bioreactor after autoclaving by connecting a bottle to the bioreactor under sterile conditions and pumping the liquid into the bioreactor.

For discontinuous fed-batch cultivation two initial glucose concentration conditions (30 g/L and 50 g/L) were tested. In both cases, the initial volume of medium in the bioreactor was 0.5 L. During the cultivation, the glucose concentration in the bioreactor was regularly measured with a blood glucose meter (Medisana MediTouch®). When the glucose concentration dropped below 30 g/L for the cultivation started with 50 g/L or when the glucose concentration dropped below 25 g/L for the cultivation started with 30 g/L, a 40% glucose solution was added to the bioreactor to reach the initial concentration of 50 or 30 g/L, respectively. This addition was necessary after 48 h of cultivation in both cases.

The first sample was taken immediately after inoculation. Additional samples were taken at regular intervals.

After 24 h of cultivation, pH was externally measured after sampling and adjusted to 5 by adding 1 M HCl if it was higher than 7. The switch to external measurement is a consequence of fungal growth on the pH probe, which compromised the accuracy of the measurement. Thus, the automatic addition of HCl was deactivated to avoid adding more acid than necessary to the bioreactor, which could lead to cell lysis.

For specified experiments, Antifoam 204 (Sigma-Aldrich) was used at a concentration of 0.01% (v/v) and sterilized by autoclaving.

### Design of experiments (DoE)

DoEs were performed with the support of the MODDE software (Sartorius). The software was used to evaluate the results and generate the response contour plots (RCP), a graphical representation of the model generated by the software. A DoE approach was used to screen the effect of the starting pH and temperature on four responses: biomass, erythritol concentration, the ratio of erythritol to biomass, and the ratio of erythritol to glucose consumed. This assay was performed with a full factorial design with two factors (starting pH and temperature) at two levels. This resulted in 15 runs. The model was fitted with multiple linear regression. The runs were performed using cultivation in shake flasks for 96 h.

A DoE approach was also used to find an optimum concentration of glucose and urea for erythritol synthesis. Two Doehlert designs were used, with 3 levels for the glucose concentrations and 5 levels for the urea concentrations. A central point was added to both designs, and an additional point was added in the first design. All combinations were tested in triplicates except the additional point tested in duplicates. This resulted in 23 runs for the first Doehlert and 21 runs for the second one. The model was fitted with partial least square regression. The runs were performed using cultivation in shake flasks for 168 h.

For all experiments, flasks were sampled every 24 h. Samples were used for pH measurement and HPLC for sugar and polyol quantification. Biomass was determined at the end of cultivation by dry weight measurement.

### Biomass determination

Biomass was determined by measuring dry weight at the end of a cultivation.

For shake flasks experiments, filtration was performed using Grade 54 quantitative filter papers hardened low-ash (Whatman®) assembled on a vacuum filtration system. The system consisted of a glass funnel, a glass base, a stopper for vacuum filtration fixed with a clamp and fixed on a Büchner Glass flask connected to a vacuum pump. Before filtration, filters were numbered, dried at 80 °C overnight, placed in a desiccator, and weighed on a precision scale. After filtration, the filters underwent the same procedure, and the biomass was calculated from the difference between the two masses. Flasks containing only the medium were used as controls.

For bioreactor experiments, bioreactors were disassembled at the end of the cultivation, and agglomerates were transferred on an aluminum foil previously weighed. The remaining biomass was separated from the medium by filtration through a piece of Miracloth (MilliporeSigma®), previously dried and weighted, and placed in a funnel. The Miracloth containing the biomass and the agglomerates on the aluminum foil were dried at 80 °C for one to three days.

### HPLC

Polyols and sugars were determined by HPLC measurement on a system equipped with a DGU-ZOA 3R degassing unit (Shimadzu), Nexera XR LC-20AD 2X liquid chromatograph pumps (Shimadzu), a SIL20AC autosampler (Shimadzu), a CTO-20A column oven (Shimadzu) and a 20A refractive index detector (Shimadzu). Two settings of guard and analytical columns were used, one with Aminex HPX-87H (Biorad) and one with Sugar SH-G and Shodex SH1011 (ShowaDenko). The HPLC method consisted of an injection volume of 10 μL, an elution with an isocratic flow of 0.6 mL/min with 5 mM sulfuric acid, and a column and detector temperature of 50 °C. The mobile phase was prepared with ultrapure water and filtered using a Steritop® 0.2 μm PES, 500 mL (MilliporeSigma®). Samples were filtered with Phenex™ RC membrane 0.2 μm 15 mm syringe filters (Phenomenex®). Quantification was done using an external standard calibration curve and manual or automatic integration. Calibration curves were determined by measuring two to three dilutions of the same level points to consider the potential variation caused by dilution in the calibration curve. Calibration curves were validated afterward by measurement of samples of known concentration, and the R^2^ values had to be above 0.99.

## Results

### *T. reesei* prefers glucose over erythritol as a carbon source

Since this study aimed to test the feasibility of using *T. reesei* to produce erythritol, it is essential to know whether *T. reesei* can consume erythritol. As a C4 sugar, this is highly likely, and therefore, carbon sources that might be preferred and thereby prevent consumption of erythritol were also tested. For this purpose, *T. reesei* was grown in shake flasks on glycerol, glucose, erythritol, and combinations of these three carbon sources, with a targeted initial carbon source concentration of about 55 mM (equivalent to 10 g/L glucose) and with 20 mM ammonium sulfate as a nitrogen source for 93.5 h. Samples were taken regularly, and analysis of erythritol, glycerol, and glucose in the supernatants was performed by HPLC, followed by the determination of the biomass at the end of the cultivation. We tested only the extracellular presence of the above-mentioned compounds as the intracellular erythritol concentration is expected to be low, and its recovery would implicate the unreasonable effort for any potential industrial process of disrupting the fungal cell.

**Fig. 1:**
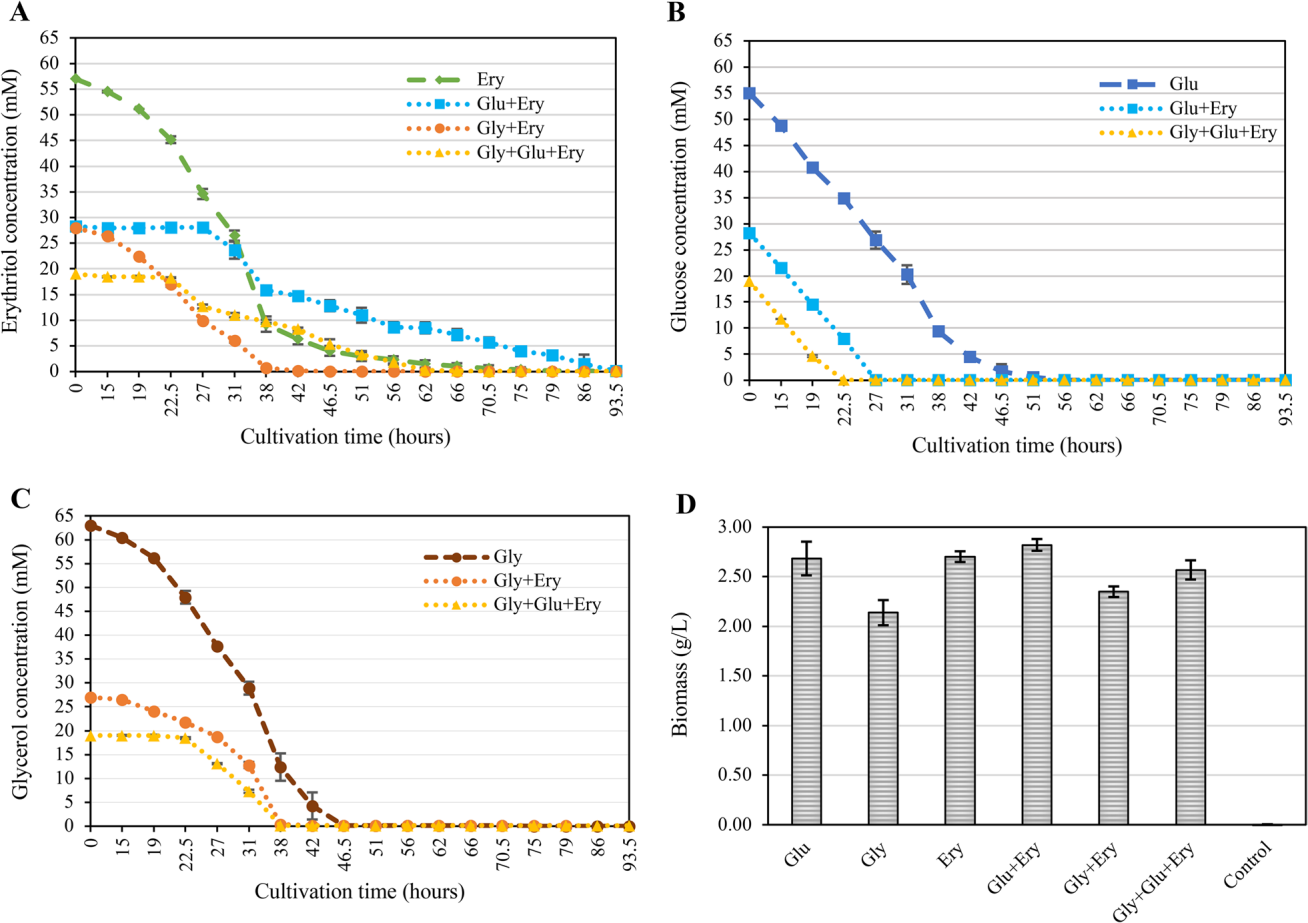
Development of carbon source concentration during cultivation of *T. reesei. T. reesei* was cultivated in shake flasks on glycerol (Gly), glucose (Glu), erythritol (Ery), glycerol and erythritol (Gly + Ery), glucose and erythritol (Glu + Ery), and glycerol and glucose and erythritol (Gly + Glu + Ery) for 93.5 h. Samples were taken at the indicated time points, and erythritol (**A**), glucose (**B**), and glycerol (**C**) concentrations were determined by HPLC in the supernatants. Biomass was determined by dry cell weight measurement at the end of the cultivation (**D**)

We observed that *T. reesei* can consume erythritol (Fig. 1A). Comparing Fig. 1A and Fig. 1B shows that when glucose is present in the medium, erythritol is consumed only after the depletion of glucose. This could be observed after 22.5 h in the condition with glucose, glycerol, and erythritol and after 27 h in the condition with glucose and erythritol. Similarly, glycerol consumption starts with glucose exhaustion (Fig. 1B and C at 22.5 h). Interestingly, glycerol and erythritol are consumed simultaneously when present together, which can be inferred by comparing the condition containing erythritol and glycerol (Fig. 1A, C). The rate of glucose consumption is consistent across all tested conditions, with identical curve slopes observed until glucose depletion (Fig. 1B). Glucose emerges as a preferred substrate over the tested polyols. It may exert a catabolic repression on polyols, constraining the expression of enzymes necessary for polyol utilization. No discernable preference is evident between erythritol and glycerol. The consumption of erythritol suggests a transport into (and out of) the cell. Biomass is comparable when glucose and erythritol are offered as carbon sources, with a slight decrease in the presence of glycerol (Fig. 1D). Considering any process development, selecting a carbon source preferable over erythritol is imperative to mitigate its consumption and maintain a concentration that reliably represses erythritol utilization by the fungus.

### Increase of glucose concentration stimulates polyol synthesis and does not affect *T. reesei* growth

**Fig. 2:**
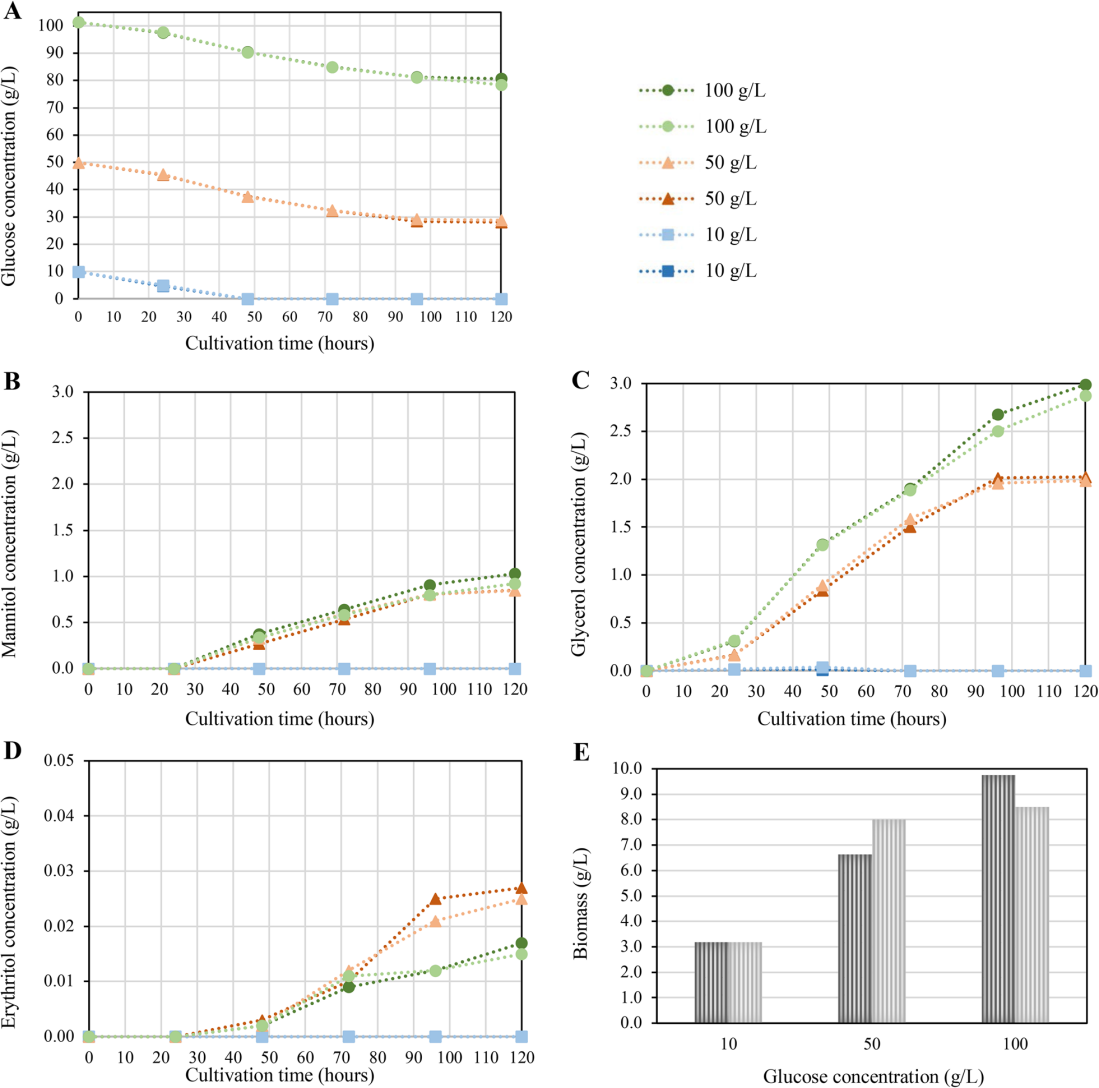
Development of polyol concentrations in dependence on the initial glucose concentration. *T. reesei* was cultivated in shake flasks on three different initial glucose concentrations, i.e., 10, 50, and 100 g/L for 120 h. Samples were taken at the indicated time points, and glucose (**A**), mannitol (**B**), glycerol (**C**), and erythritol (**D**) concentrations were determined by HPLC in the supernatants. Biomass was determined by dry cell weight measurement at the end of the cultivation (**E**). The experiment was performed in biological duplicates, and values are displayed for all replicates.

A high glucose concentration is required for erythritol production in yeasts, mainly attributed to the resulting osmotic stress [27]. To test whether this is beneficial for erythritol synthesis in *T. reesei* and whether the growth of the fungus might be impaired at high glucose concentrations, tests in shake flasks were performed with three concentrations of glucose, i.e., 10 g/L (which is the standard for growing *T. reesei*), 50 g/L and 100 g/L, and 20 mM ammonium sulfate as nitrogen source each. Carbon–nitrogen ratios for this and later described experiments can be found in Additional Table 1. This cultivation was performed for 120 h with regular sampling for the HPLC measurement of glucose and polyol concentrations, and biomass was determined at the end of the cultivation (Fig. 2).

The curves representing the glucose concentration over time have a similar pattern for all concentrations, indicating that glucose concentration decreased at a similar rate if 100 g/L and 50 g/L glucose were used, as well as during the initial 40 h if 10 g/L were used (Fig. 2A). When 50 and 100 g/L glucose were applied, glucose was still present at the end of cultivation, and glucose concentration reached a stable level, indicating that fungal growth was halted due to the limitation of a compound other than glucose or any other parameter. The glucose concentration during the cultivation decreased by about 20 g/L if the initial glucose concentration was 50 and 100 g/L (Fig. 2A).

None of the three polyols were detected during growth on an initial glucose concentration of 10 g/L (Fig. 2B–D). Still, all three polyols were detected when the initial glucose concentration was 50 g/L or 100 g/L. Specifically, the polyol concentration, when comparing 50 g/L to 100 g/L initial glucose concentration, was similar for mannitol (Fig. 2B), increased by 50% for glycerol (Fig. 2C), and decreased by 38% for erythritol (Fig. 2D). Glycerol was already detected after 24 h of cultivation (Fig. 2C), while mannitol and erythritol were detected after 48 h (Fig. 2B and D). The increase of mannitol and glycerol concentrations decelerated after 96 h, whereas for erythritol, the deceleration was only observed after 72 h when using 100 g/L glucose (Fig. 2B–D).

Notably, *T. reesei* could grow at all tested glucose concentrations; even the unusually high concentrations did not negatively impact the biomass but did result in higher biomass formation (Fig. 2E). Altogether, it can be stated that varying the glucose concentration influences polyol formation. While a concentration of 50 g/L is already sufficient for the best erythritol synthesis, an increase to 100 g/L still enhances the synthesis of glycerol and mannitol. As erythritol concentration in the supernatant increases until 120 h at least (Fig. 2D), we could hypothesize that cultivation needs to be pursued for at least 120 h, and that the initial glucose concentration should be above 20 g/L to avoid glucose depletion during this time.

### Glucose is necessary for erythritol synthesis

As described earlier, this study aimed to identify alternative feedstocks for erythritol production. For this reason, we tested whether and to which extent erythritol is synthesized by *T. reesei* when grown on three different carbon sources. The selection of the carbon sources was based on their relevance to potential alternative substrates: xylose, the primarily derived sugar from hemicellulose degradation of lignocellulosic residues; glucose, the primarily derived sugar from cellulose degradation of lignocellulosic residues and a known substrate for erythritol production; and lactose, a major component of residues from the dairy industry [28]. Concentrations were chosen to reflect the molarity of the tested glucose concentrations, i.e., 55.5 mM corresponds to 10 g/L of glucose, and 277.5 mM corresponds to 50 g/L. The first represents the standard cultivation condition for *T. reesei*, while the second represents a condition that we found to stimulate polyol production. The nitrogen source was ammonium sulfate at 20 mM. Samples were regularly taken to measure polyol and carbon source concentrations by HPLC, and biomass was measured at the end of cultivation.

**Fig. 3:**
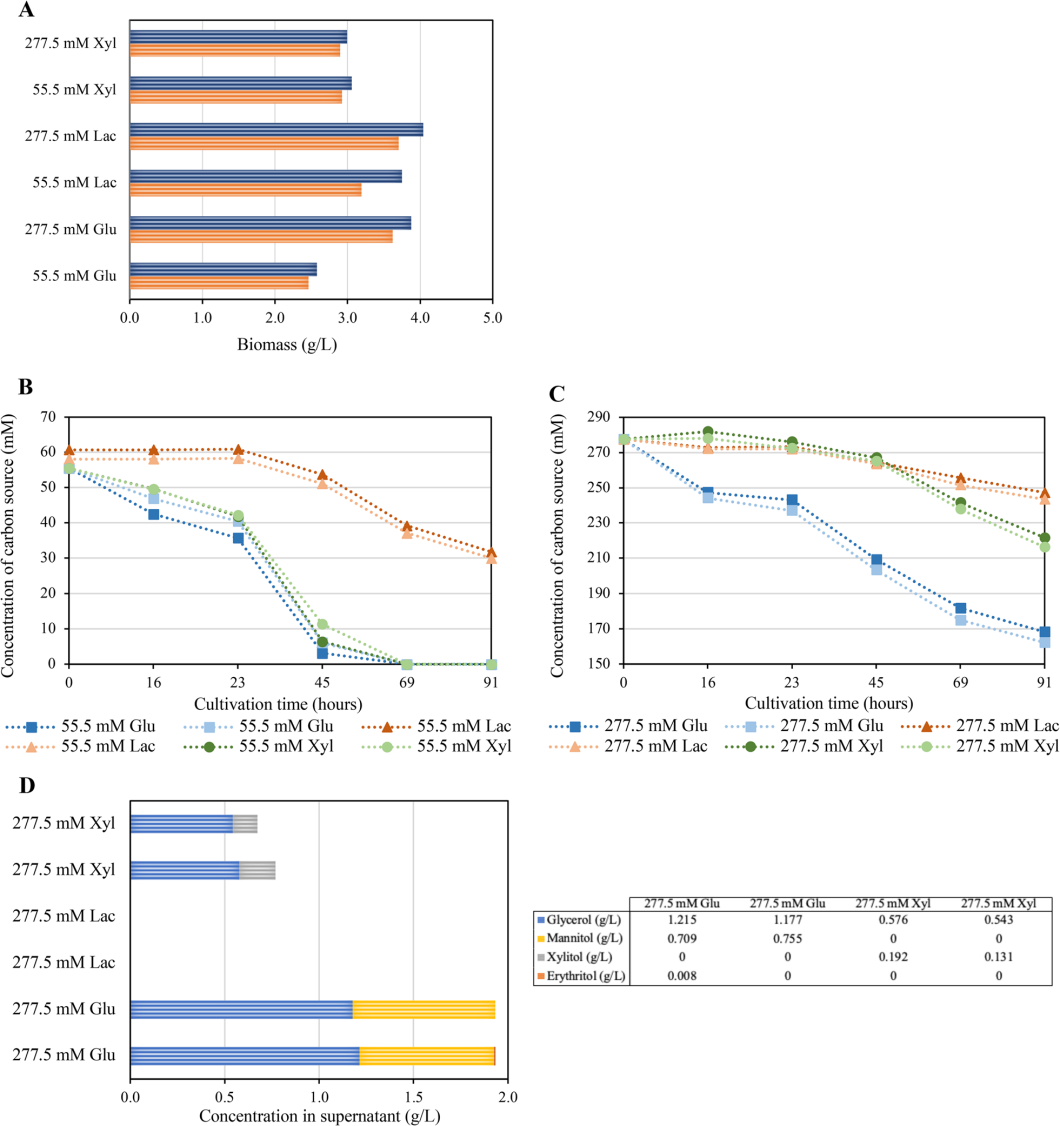
Development of polyol concentrations in dependence on the type of carbon source and its concentration. *T. reesei* was cultivated in shake flasks on three different carbon sources, i.e., [lactose (Lac), xylose (Xyl), and glucose (Glu)] at two concentrations (55.5 mM and 277.5 mM) each for 96 h. Biomass was determined by dry cell weight measurement at the end of cultivation (**A**). Samples were taken at the indicated time points, and glucose, lactose, and glycerol concentration was determined by HPLC in the supernatants of cultivations with an initial carbon source concentration of 55.5 mM (**B**) and 277.5 mM (**C**). The concentration of polyols was measured at the same time points, but for easier visualization, only the concentrations in the supernatant at the end of cultivation, which was always the highest obtained concentration, are given (**D**). The experiment was performed in biological duplicates, and values are displayed for all replicates

*Trichoderma reesei* grew on all three carbon sources (Fig. 3A).

In the case of glucose, an increase in the initial concentration resulted in higher biomass, which was less pronounced in the case of lactose and unaffected in the case of xylose (Fig. 3A). At initial concentrations of 55.5 mM, glucose and xylose were consumed without any lag time until total depletion, while lactose consumption was detected only after 23 h, and lactose was not fully consumed within the tested time frame (Fig. 3B). At the higher tested concentration, xylose and lactose consumption started after 23 h, whereas glucose consumption commenced immediately (Fig. 3C). The amount of xylose consumed during cultivation was similar regardless of whether the starting concentration was 55.5 mM or 277.5 mM, and the same was observed with lactose. In contrast, in the case of glucose, an increase in concentration led to enhanced consumption (compare Fig. 3B and C).

At an initial concentration of 55.5 mM, no polyols were detected in the supernatant for all three tested carbon sources, as expected (data not shown). At an initial concentration of 277.5 mM, already after 24 h of cultivation polyols could be detected in the supernatant dependent on the carbon source. Glucose resulted in the formation of glycerol and mannitol with trace amounts of erythritol. Xylose led to the detection of glycerol and xylitol but not of erythritol. Lactose, however, did not result in the detection of any polyols (Fig. 3D). To summarize, different types and amounts of polyols were obtained depending on the carbon source used. We decided to use glucose for further experiments because erythritol was only obtained from glucose amongst the tested carbon sources.

### The nitrogen source impacts erythritol synthesis

To determine the influence of the nitrogen source on polyol synthesis, *T. reesei* was cultivated in shake flasks with four different nitrogen sources at two concentrations each. Ammonium, nitrate, and urea were tested at 20 and 80 mM, and yeast extract (YE) was tested at 2 and 8 g/L. For comparability, the same carbon source was used, i.e., glucose at a concentration of 50 g/L, and cultivation was performed for 96 h.

**Fig. 4:**
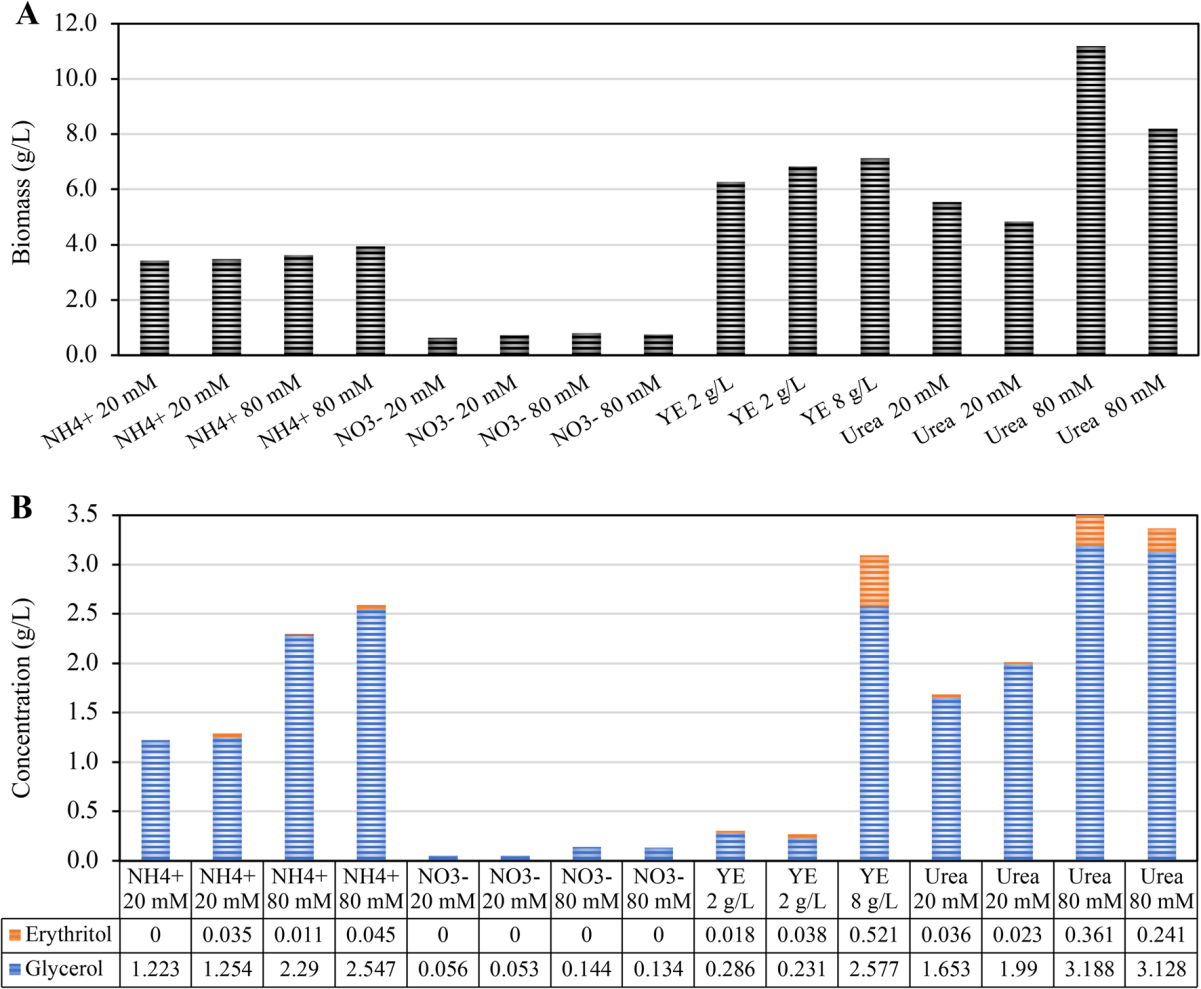
Biomass and polyol concentrations in dependence on the type of nitrogen source and its concentration. *T. reesei* was cultivated in shake flasks on 50 g/L glucose as the sole carbon source combined with four different nitrogen sources for 96 h. Ammonium (NH4+), nitrate (NO3−), and urea were added at initial concentrations of 20 mM and 80 mM, and yeast extract (YE) was added at initial concentrations of 2 and 8 g/L. Biomass was determined by dry cell weight measurement at the end of cultivation (**A**). Concentration of polyols was measured at several time points during the cultivation, but for easier visualization, only the concentrations in the supernatant at the end of cultivation, which was the highest obtained concentration, are given (**B**)

Nitrate yielded lower biomass than ammonium, while urea and YE resulted in a biomass increase of approximately 1.5 to twofold compared to ammonium (Fig. 4A).

With all nitrogen sources tested, increasing the nitrogen concentration led to higher total polyol concentrations (Fig. 4B). With ammonium and urea, the concentration increase was about 1.5 to twofold. For nitrate, despite its increase, the polyol concentrations remained low, more than 4 times lower than the total concentration of polyols obtained with 20 mM ammonium (Fig. 4B). The most significant change was observed with increased YE concentration, which resulted in a tenfold increase in erythritol concentration. The nitrogen source also impacted the concentration of each polyol independently. In the case of ammonium sulfate, the erythritol concentration remained below 0.05 g/L regardless of the used concentration. In contrast, in the case of YE and urea, the increase in the nitrogen concentration led to a tenfold increase in the erythritol concentration. In the case of YE, this tenfold increase was observed for erythritol and glycerol, whereas in the case of urea, glycerol only doubled (Fig. 4B).

Concerning the type of nitrogen source, similar glycerol concentrations were obtained, measuring 2.6 g/L in the case of 8 g/L YE compared to 2.5 and 2.3 g/L in the case of 80 mM ammonium. Otherwise, the erythritol concentration was ten times higher with 8 g/L YE compared to 80 mM ammonium.

Altogether, the nitrogen source exerts a notable influence on biomass, total polyol concentration in the supernatant, and the distribution of different polyols (Fig. 4B). For subsequent experiments, the best-identified condition, i.e., the usage of 80 mM urea as the nitrogen source, was applied.

### Erythritol concentration is mainly impacted by urea concentration, while glucose has a limited impact

After identifying a preferable carbon and nitrogen source for erythritol synthesis, we wanted to find an optimal combination of both parameters. For this purpose, we used DoE. Based on previous tests, we defined the range of glucose concentration to study between 40 and 90 g/L and the range of urea concentration from 20 to 100 mM. We selected a Doehlert design, which is a reduced optimization design because we assumed the absence of interaction between glucose and urea. We suspected that glucose would have a limited effect if maintained at a high enough concentration to prevent erythritol consumption. We performed the Doehlert design twice. In both assays, we used two factors, namely urea, and glucose, with three levels for glucose (varying from design) and five for urea (20 mM, 40 mM, 60 mM, 80 mM, and 100 mM), each combination tested in triplicates with a central point tested in triplicates. Two responses were measured, i.e., the biomass at the end of cultivation and the erythritol concentration in the supernatant. The design was first performed with the glucose levels 40 g/L, 60 g/L, and 80 g/L with a central point at 60 g/L and 60 mM urea and an additional point at 50 g/L and 80 mM urea (Additional Fig. 1A). The second design was performed with the glucose levels 50 g/L, 70 g/L, and 90 g/L and a central point 71 g/L glucose and 60 mM urea (Additional Fig. 1B). The RCPs obtained are displayed in Additional Fig. 1. The model indicates that within the tested concentration range of 40 to 90 g/L, glucose concentration has a limited effect on erythritol synthesis; only the urea concentration exhibits an influence. Biomass, however, is influenced by the glucose concentration (Additional Fig. 1). The optimal conditions identified by this DoE were subsequently employed for further experimentation. During this experiment, it was observed that using urea resulted in elevated pH. Consequently, an evaluation of the impact of the initial pH and temperature on both biomass formation and erythritol synthesis was conducted.

### Up-scaling from shake flasks to bioreactors

To test whether the conditions identified in shake flasks are transferable to the bioreactor scale, we used the previously identified optimum concentration of glucose and urea. We tested the parameters stirring speed, impeller type, aeration, and operation mode. All tested configurations are displayed in Table 1 together with the obtained results.

**Table 1:** Overview on the conditions tested in a bioreactor

No	*V* (L)	*T* (°C)	Imp*	Stir (rpm)	Aer (vvm)	Op mode	AF	Cult time (h)	Biomass (g/L)	Ery 48 (g/L)	Ery f (g/L)	Man (g/L)	Gly (g/L)	Glu cons (g/L)	Glu add (mL)	E/Glu (mg/g)	E/B (mg/g)	B/Glu (g/g)
1	1.25	30	M(t)R (b)	700	0.6	B 70	No	48	23.0	0.16	0.16	0.49	1.40	32.5	–	4.9	7.0	0.71
2	1	30	R(t)R (b)	400	0.79	B 70	No	48	23.9	0.36	0.36	0.90	1.37	46.4	–	7.8	15.1	0.51
3	0.5	28	M (b)	300	0.5	B 70	Yes	72	18.1	0.10	0.17	0.00	1.28	20.0	–	8.5	9.4	0.91
4	0.5	28	M (b)	400	1.6	B 70	Yes	72	23.1	0.11	0.23	0.24	2.79	27.2	–	8.4	10.0	0.85
5	0.5	28	M (b)	300	0.5	B 70	No	72	13.7	0.09	0.18	0.18	1.44	22.8	–	8.0	13.1	0.60
6	0.5	28	M (b)	300	0.5	B 70	No	96	19.0	0.06	0.31	0.81	0.80	40.6	–	7.6	16.3	0.47
7	0.5	28	M (b)	300	0.5	FB 50	No	96	11.1	0.05	0.11	0.00	0.39	16.2	25	6.8	9.9	0.68
8	0.5	28	M (b)	300	0.5	B 70	No	72	13.0	0.09	0.17	0.19	0.94	19.9	–	8.5	13.1	0.65
9	0.5	28	M (b)	300	0.5	FB 30	No	72	10.8	0.14	0.18	0.89	0.68	20.0	9	9.0	16.7	0.54

No: number of experiment; *V*: volume of medium used; *T*, temperature; Imp, impeller type; M: marine blade; R: Rushton; (b), positioned in the bottom half of the bioreactor; (t), positioned in the top half of the bioreactor; Stir, stirring speed of the impeller; Aer, aeration expressed in vvm [airflow rate (L/min)/volume of culture broth (L)]; OP mode, operational mode; B, batch; FB, fed-batch; 70, start with a glucose concentration of 70 g/L; 50, start with a glucose concentration of 50 g/L, 30, start with a glucose concentration of 30 g/L; AF, presence of antifoam; Cult time, time at which the cultivation was stopped; Ery 48, concentration of erythritol in the culture broth after 48 h of cultivation; Ery f, concentration of erythritol in the culture broth at the end of cultivation; Man, concentration of mannitol in the culture broth at the end of cultivation; Gly, concentration of glycerol in the culture broth at the end of cultivation; Glu cons, total glucose consumed during cultivation; Glu add, volume of 40% glucose solution added after 48 h of cultivation; E/Glu, ratio of formed erythritol to consumed glucose; E/B, ratio of formed erythritol to formed biomass; B/Glu, ratio of formed biomass to consumed glucose

^*^When the volume was reduced to 0.5 L, only one impeller could be used due to the reduced volume

Cultivation in bioreactors led to biomass formation of up to threefold higher than in shake flasks. In only 48 h 23 g/L biomass was obtained in bioreactors compared to 10 g/L in shake flasks after 5 days. This strong growth in the bioreactor—accompanied by foaming—concerned bioreactor elements like sparger, probes, and outlet air filters leading to a build-up of pressure in the bioreactor. In such cases, cultivations had to be stopped due to safety reasons, but not due to low glucose concentrations (see Table 1).

Several bioreactor setups were tested to decrease this uncontrolled growth and foaming by varying the impeller type, stirring speed, and aeration rate. Still, none of these setups allowed a cultivation time longer than 48 h. Amongst these initial experiments, the results of two cultivations are provided in Table 1 (no. 1 and 2). It turned out that an extension of the cultivation time was only possible by reducing the cultivation volume (Table 1, no. 3 to 10). This prevented that the fungus reached the top of the bioreactor and would eventually clock air filters and build-up pressure. We also decided to use a marine blade instead of Rushton turbine to limit foaming and to decrease the temperature to 28 °C, as a temperature of 28 °C in a bioreactor is closer to the temperature inside a shake flask when incubated in an incubator at 30 °C (Table 1, no. 3–10).

Further addressing foaming concerns, a bioreactor with antifoam was set up (experiment no. 3). The cultivation could be extended to 72 h, the biomass formation was slightly lower than in the cultivation no. 1 and 2 without antifoam, and the erythritol concentration was lower than in cultivation no. 2, but higher than in no. 1.

To test whether aeration influences biomass formation and erythritol production, we performed a cultivation using an aeration of 1.6 vvm and slightly higher stirring (Table 1, no. 4). Antifoam was kept because we suspected that increased aeration might increase foaming again. In the cultivation with the higher aeration more biomass and polyols were formed and more glucose was consumed. However, the ratios of erythritol to glucose consumed and erythritol to biomass were similar (Table 1). This suggested that aeration favors biomass formation while having a lower impact on erythritol formation.

Next, we tested the effect of the antifoam by cultivating in the same conditions as in cultivation no. 3, but without antifoam (no. 5). Biomass was 32% higher with antifoam; however, the erythritol concentration was in a similar range (Table 1).

Glucose fed-batch was tested because it might lower the growth and thereby, the viscosity of the medium, thus, also reducing uncontrolled growth and foaming. Two experiments using two bioreactors each, a control (batch) cultivation and a fed-batch cultivation inoculated with the same pre-culture, were performed. In the first experiment, the control cultivation had an initial glucose concentration of 70 g/L (no. 6), and the fed-batch cultivation had an initial glucose concentration of 50 g/L (no. 7). In the second experiment, the control cultivation was identical (Table 1, no. 8), and the fed-batch cultivation had an initial concentration of 30 g/L (no. 9).

The cultivation could be prolonged to 96 h in case of the cultivations no. 6 and 7. For both fed-batch cultivations, the biomass formed was lower than in their respective control. Regarding erythritol formation and glucose consumption, the two fed-batch cultivations had different behaviors, the fed-batch at 30 g/L had the best erythritol to biomass and erythritol to glucose consumed yields, but the benefit of the fed-batch is not clearly pronounced.

The cultivation in bioreactors did not reach the concentration observed in shake flasks of 0.8–1 g/L erythritol, nor did they allow us to clearly identify a positive effect of a fed-batch cultivation, the addition of antifoam, or any other tested parameter. At the same time, we could not satisfactorily solve the uncontrolled growth in the bioreactors. Consequently, we decided to test the effect of pH and temperature again in shake flasks.

### The starting pH and the incubation temperature impact extracellular erythritol concentration through an interaction when urea is the nitrogen source

**Fig. 5:**
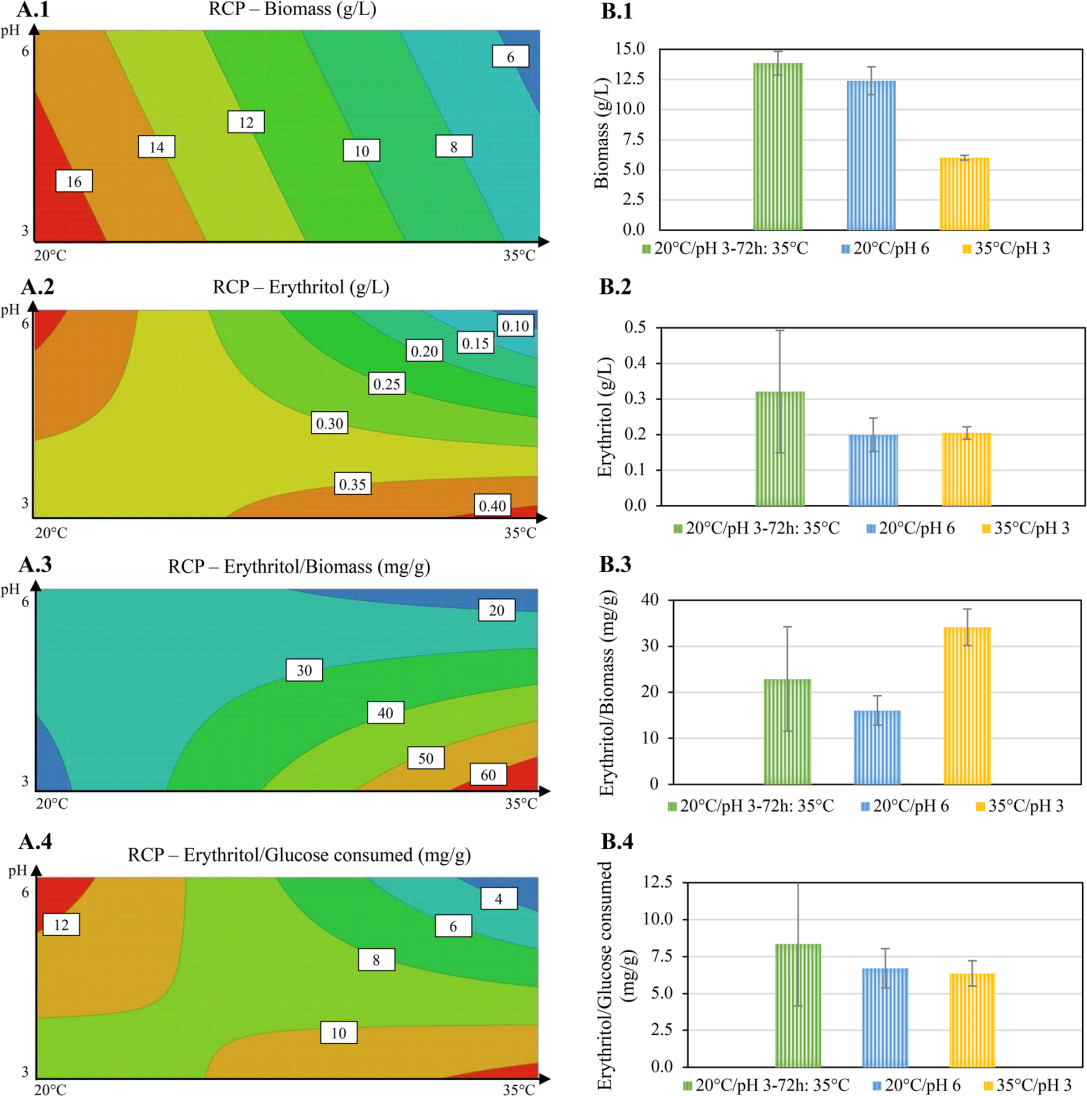
Results and verification of the DoE on the impact of pH and temperature. *T. reesei* was cultivated in shake flasks on 70 g/L glucose as the sole carbon source and 80 mM urea as the nitrogen source for 96 h. A full factorial DoE with 2 levels was performed with temperatures 20 °C and 35 °C, and pH 3 and 6. Cultivations were performed in triplicates. Samples were taken every 24 h, and glucose and erythritol concentrations were determined by HPLC in the supernatants. Biomass was determined by dry cell weight measurement at the end of cultivation. The results were used to generate a model illustrated by RCPs for biomass (**A.1**), erythritol concentration (**A.2**), the ratio of erythritol concentration to biomass (**A.3**), and erythritol concentration to glucose consumed (**A.4**). The suggested extreme conditions (20 °C at pH 3 and 35 °C at pH 6 for 96 h of cultivation) and an additional condition (20 °C at pH 3 for 72 h followed by 24 h at 35 °C) were applied and the same parameters, namely biomass (**B.1**), erythritol concentration (**B.2**), the ratio erythritol concentration to biomass (**B.3**), and the ratio erythritol concentration to glucose consumed (**B.4**), were measured and calculated

For the above-mentioned reason, a DoE was performed to investigate the correlation between the starting pH and temperature and to evaluate their impact on four responses: biomass, erythritol concentration, the ratio of erythritol to biomass, and the ratio of erythritol to glucose consumed. The obtained RCPs are depicted in Fig. 5A. The biomass formation is predominantly influenced by the temperature: within the temperature range of 20 °C to 35 °C, lower temperatures favor biomass formation, while the effect of the starting pH on growth remains consistent at both low and high temperatures (Fig. 5A.1). At a given temperature, a starting pH close to 3 is preferable to a starting pH close to 6 to generate biomass (Fig. 5A.1).

Erythritol is affected by an interaction between temperature and starting pH (Fig. 5A.2). This is visible by the symmetry of the plot. It indicates that the starting pH affects erythritol concentration differently based on temperature. At 20 °C, a pH between 5.5 and 6 results in erythritol concentrations exceeding 0.4 g/L, while at temperatures between 33 and 35 °C, a pH of 3 yields a similar concentration (Fig. 5A.2).

When the data are analyzed regarding the ratio of erythritol concentration to biomass, the best condition is high temperature (35 °C) and low starting pH (Fig. 5A.3). This condition combines the highest erythritol concentration with low biomass, which explains the high ratio. The best conditions for the ratio of erythritol concentration to glucose consumed are the same as the ones generating the highest erythritol concentration: 35 °C/pH 3 and 20 °C/pH 6 (Fig. 5A.4).

To validate the obtained predictions, an additional experiment employing three conditions was conducted: it comprises the two best-predicted conditions for erythritol production, i.e., 20 °C/pH 6 and 35 °C/pH 3 incubated for 96 h, and an incubation for 72 h under the optimal condition for biomass formation (20 °C/pH 3) followed by an incubation for 24 h at the optimal condition for erythritol production (35 °C/pH 3) (Fig. 5B).

The results demonstrate that the model is predictive of the formation of biomass. However, for erythritol synthesis, the model can predict a trend but not values (Fig. 5B). The biomass obtained in the experiment to test the model aligns with model predictions (Fig. 5B.1): the obtained biomass was lower for the condition 35 °C/pH 3 than for the condition 20 °C/pH 6, and the values are similar to the expected values (i.e., 0.6 g for the condition 35 °C/pH 3 and 1.4 for the condition 20 °C/pH 6). This is close considering the standard deviation of the formed biomass. For erythritol synthesis, the conditions 20 °C/pH 6 and 35 °C/pH 3 gave similar numbers as expected, but the concentration was twice lower than expected, i.e., 0.2 g/L obtained for an expectation of at least 0.4 g/L (Fig. 5B.2). The same was observed for the ratio of erythritol to biomass and of erythritol to glucose consumed: the values obtained followed the trend of the prediction, but the ratios obtained were half of those predicted by the model (Fig. 5A.3 and A.4).

The tested condition with a temperature change after 72 h showed a slightly higher biomass (1.385 g ± 0.098 g), but not significantly different, compared to the condition 20 °C/pH 6 (Fig. 5B.1), and a higher erythritol concentration, but with also a higher standard deviation (Fig. 5B.2). Thus, it is difficult to conclude whether this condition will lead to better erythritol yield.

## Discussion

### *T. reesei* consumes erythritol—consequences for a potential process

As found during this study, *T. reesei* synthesizes erythritol when the glucose concentration exceeds 30 g/L and metabolizes it when no preferred carbon source is available. Therefore, any cultivation medium must constantly contain a carbon source preferred over erythritol to prevent its consumption. For example, given that *T. reesei* does not prioritize glycerol over erythritol as a substrate, means that glycerol cannot be employed as a carbon source for the erythritol production phase. However, it may be useful for biomass generation. As the chosen carbon source has to be present in the media until the end of the cultivation to avoid erythritol consumption, it implies that this carbon source will be present in the final culture broth. Consequently, the recovery process must enable an effective separation of erythritol and the chosen carbon source. An alternative approach would be the extraction of erythritol during the process to stimulate its formation and prevent consumption. However, this may require membrane technology, making it a potentially expensive and challenging process [29].

It is crucial to note that not all carbon sources lead to erythritol production. Erythritol could be obtained solely in the presence of glucose, with a maximum concentration of 1 g/L in the supernatant reached after seven days, in contrast to yeasts and yeast-like fungi that can produce 1 to 3 g/L/h [8, 9, 30]. In addition, *T. reesei* produces mannitol and glycerol in significantly higher concentrations than erythritol, posing challenges for a potential downstream process as the separation of erythritol and glycerol is intricate due to their close structural and property resemblance.

### Comparison to the existing yeast-based process

*T. reesei* has amongst the tested carbon sources a preference for glucose as a carbon source for erythritol production. Glucose is also in the yeast-based processes the used carbon source. While the possible carbon sources for growing yeasts is generally restricted compared to *T. reesei* due to their limitations to metabolize C5-sugars, in terms of yields and productivity, yeasts naturally produce better than *T. reesei.* For example, screening of *M. pollinis* isolates revealed wild-type strains that able to produce concentrations up to 111 g/L in 160 h of cultivation, using an initial glucose concentration of 300 g/L [31]. Glycerol has been reported to lead to erythritol production in *Yarrowia lipolytica;* however, this yeast can also consume erythritol decreasing the yield of the process [27, 32–34]. The same was observed in this study for *T. reesei*. So far, no reports exist about the ability of yeasts to produce erythritol from lactose. In this study no polyols could be detected when *T. reesei* was grown on lactose. Regarding xylose, reported processes have been tested with mutant strains. An *A. pullulans* mutant could produce 0.22 g/L/h erythritol from an initial xylose concentration of 120 g/L [35, 36]. So far, no reports exist about the natural ability of yeasts to produce erythritol from xylose. Also, *T. reesei* grown on xylose does not synthesize erythritol, but other polyols.

### Difficulties during scale-up

Another challenge with *T. reesei* in comparison to yeasts is the cultivation in a liquid medium. This became obvious in this study as some of the tested conditions led to high fungal growth, which was particularly evident with the substantial increase in biomass formation upon the addition of 80 mM urea (Fig. 4). This fast growth was more pronounced in bioreactors, likely due to higher aeration, leading to high biomass production rather than erythritol formation. The observed growth of *T. reesei* on the bioreactor’s probes and its internal surface is a known phenomenon in filamentous fungal cultivations, usually reported for extended cultures [37]. In our study this was observed after 48 h, which could be attributed to the concentration of carbon and nitrogen sources, which was a lot higher than in standard *T. reesei* cultivation conditions (usually around 10 g/L carbon source and 20 mM nitrogen source) [26]. The tested strategies of reducing aeration, stirring and volume, allowed us to extend the cultivation time. We aimed for the later because in the shake flasks cultivation times of at least 72 h allowed to obtain erythritol concentrations of above 0.3–0.5 g/L. However, in the case of the bioreactor cultivations the increased time did also not lead to higher erythritol concentration.

Alternatively, to address the observed uncontrolled growth, a separation of the growth and erythritol production phases could be used. Such a strategy would additionally allow the usage of different waste streams in a biorefinery approach. It will offer the possibility to make use of the ability of *T. reesei* to grow on various carbon sources without significant impact on biomass, opening avenues for utilizing xylose, lactose, or glycerol-based media during the growth phase. Following the growth phase, a medium exchange could be performed, exposing the fungus to conditions favoring erythritol synthesis, i.e., a medium with a glucose concentration above 30 g/L and yeast extract or urea as nitrogen source. Investigating the effect of nitrogen sources on growth separately from the erythritol production phase could bring additional improvements. Also, further parameters could be investigated, such as exploring the effect of osmotic stress types. Exploring the osmotic stress types contributing to erythritol production could enable substituting a part of the glucose with another solute. However, any selected solute must not compromise downstream processes.

### Successes and limits of the DoE approach

In this study, the models obtained by DoE were predictive for patterns and offered insights into how certain conditions might perform. However, quantitative predictions, especially for erythritol, were not precise, primarily due to system variation. DoE approaches are highly sensitive to variation, encompassing process, analytical, and unknown variations. Process variation, regarded as "execution variation", includes subtle differences in volumes or used quantities created by the operator. Analytical variation stems from the analytical tools used, and the unknown variation represents the remaining variation linked to non-controlled parameters. The dry cell weight determination method for biomass was optimized, yielding a standard deviation below 5%, rendering the model reliable. However, analytical methods for polyols exhibited stronger variation. Glycerol and mannitol variations were below 10%, while erythritol variation was around 20%, likely due to the small quantity measured. This 20% variation, together with process and unknown variations (estimated at least at 10%), results in a variation of over 30%. This means that if an effect on erythritol can be detected, a parameter would need to increase or decrease the extracellular concentration of erythritol by more than 30%. This example explains why the high sensitivity of DoE to variation poses challenges in early process stages when processes are often not yet robust. Therefore, developing robust analytical methods is a pre-requisite for early stage DoE investigations to keep the overall variation as low as possible, as the process variation itself is high at this point.

### Export of polyols or cell lysis

Polyols were measured in the supernatant, which could be the result of an export out of the cell but also raise the possibility of cell lysis. However, no significant cell lysis was observed, and the consistent increase in polyol concentration in the supernatant contradicts an abrupt lytic release. The consumption of erythritol by *T. reesei* further implies the presence of an erythritol transporter. Current knowledge about polyol transporters in filamentous fungi is limited. Some transporters have been identified in yeasts and bacteria, but remain poorly characterized [38, 39].

### Influence of carbon and nitrogen availability

Investigation on the influence of the carbon and nitrogen source includes considering three aspects: the nature of the carbon or nitrogen source, the concentration of the carbon or nitrogen source and the ratio of both (C/N ratio). The results of the DoE (Additional Fig. 1) pointed at an influence of the nitrogen source rather than at the influence of the C/N ratio. Ratios have been, however, calculated and can be found in Additional Table 1. In the assay testing different glucose concentration, the best erythritol production (see Fig. 2) was obtained with a C/N ratio of 83 (Additional Table 1). However, in the assay testing different nitrogen sources, nitrate and ammonium at the same C/N ratio of 83 and led to different erythritol production (compare Additional Table 1 and Fig. 4). For urea and yeast extract the erythritol production was higher (Fig. 4), while the C/N ratio was lower at around 10 or 20. This does not point at an impact of the C/N ratio, but rather at an impact of the nature of the nitrogen source itself. In addition, if the C/N ratio would play a major role, it would have been observed in the symmetry of the response contour plot (Additional Fig. 1). Combinations corresponding to similar C/N ratio would have led to similar erythritol concentrations, which would result for these points being in areas of the same color. This means the points corresponding to the combinations 50 g/L glucose and 40 mM urea, would have been in the same color area as 75 g/L glucose and 60 mM urea. Importantly, this was not the case. The DoE results pointed at a strong impact of the urea concentration independently from the glucose concentration in the tested domain, which does not support an impact of the C/N ratio.

### Absence of polyols using lactose as carbon source

When using lactose as carbon source, no polyols were measured in the supernatant. There are no reports about the possibility of producing erythritol from lactose in yeasts or filamentous fungi. However, we would have expected the detection of some polyols such as galactitol or sorbitol (lactitol is not a natural polyol, it is formed by catalytic hydrogenation). The employed HPLC method was optimized for the detection of mannitol, erythritol, xylitol, and glycerol. Any other polyol, such as galactitol, could have been formed, but not detected because its concentration was below the detection limit of the method. Sorbitol and galactitol could also have been formed but not released extracellularly. Another hypothesis is that the cultivation time was too short. We observed in the case of glucose that different polyols started to be detected at different times, for example, glycerol was already detectable after 20 h of cultivation, whereas erythritol and mannitol were detected only after 48 h (Fig. 2). Lactose started to be consumed a lot later than glucose (between 23 and 45 h of cultivation). Thus, if polyols would be produced, they likely would not appear before 71–93 h of cultivation.

### Absence of erythritol using xylose as carbon source

**Fig. 6:**
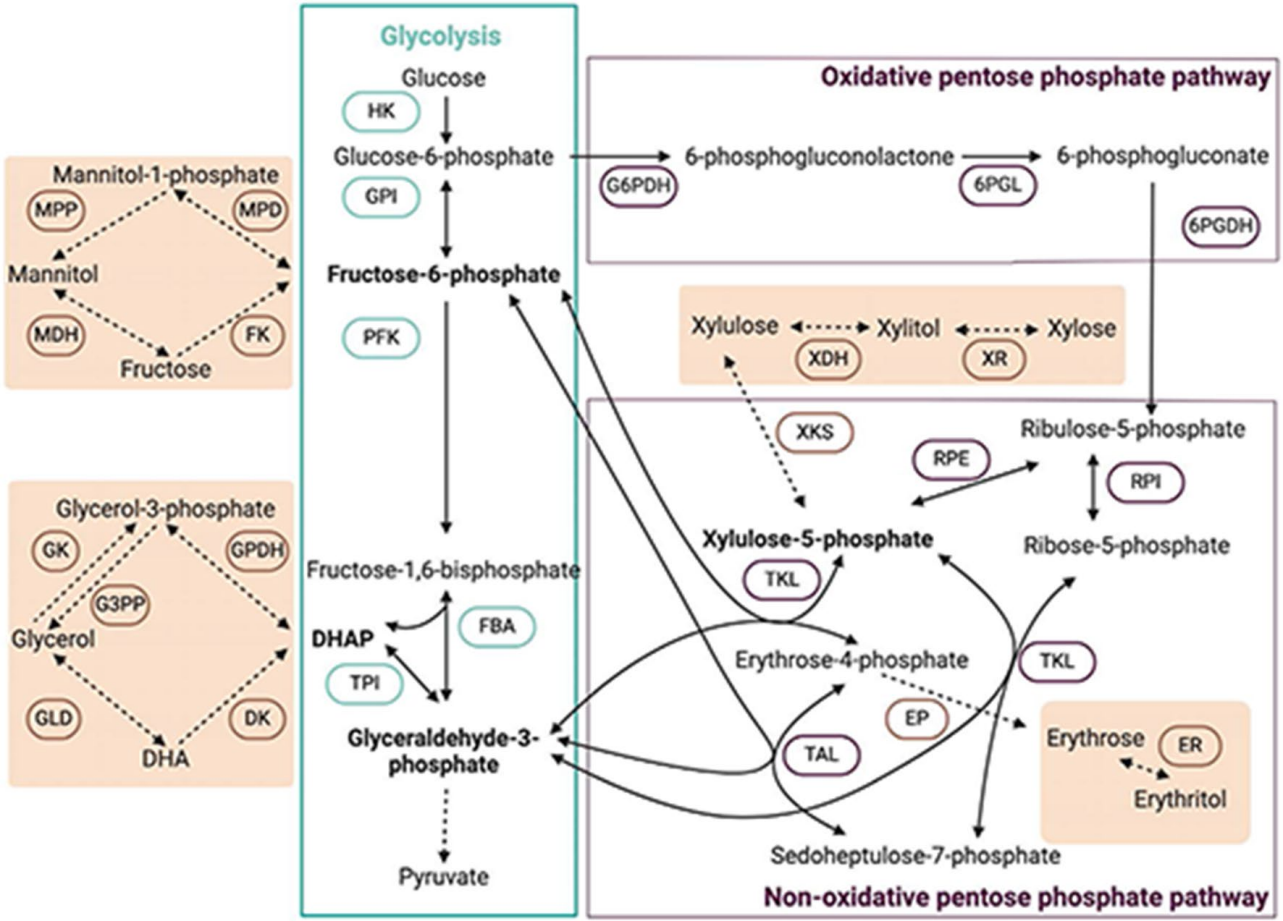
Connection of mannitol and glycerol metabolism to glycolysis and the pentose phosphate pathway. Dashed arrows indicate reactions in which the direction or the attributed enzyme is not fully confirmed. HK: Hexokinase; GPI: Phosphoglucoisomerase; PFK: Phosphofructokinase; FBA: fructose bisphosphate aldolase; TPI: triose phosphate isomerase, DHAP: dihydroxyacetone phosphate, GPDH: glycerol phosphate dehydrogenase, DK: Dihydroxyacetone kinase; GLD: glycerol dehydrogenase; GK: glycerol kinase; G3PP: Glycerol-3-phosphate phosphatase; MPD: mannitol phosphate dehydrogenase; MPP: mannitol phosphate phosphatase; MDH: mannitol dehydrogenase; FK: fructokinase; XR: xylose reductase; XDH: xylitol dehydrogenase; XKS: xylulose kinase; TKL: transketolase; RPE: ribulose-5-phosphate-3-epimerase, RPI: ribose-5-phosphate isomerase, TAL: transaldolase, EP: Erythrose-4-phosphate kinase, ER: erythrose reductase, G6PDH: glucose-6-phosphate dehydrogenase, 6PGL: 6-phosphogluconolactonase, 6PGDH: 6-phosphogluconate dehydrogenase

When using xylose as carbon source, no erythritol was detected. Synthesis of erythritol by *T. reesei* from xylose was previously described, but the concentration was only 70 µg/g of mycelium, and erythritol was found only in the mycelium [21]. Otherwise, the production of xylitol and glycerol from xylose can be explained by the current knowledge of xylose assimilation in fungi (Fig. 6). Upon entering the fungal cell, xylose undergoes conversion to xylitol and subsequently to xylulose and xylulose-5-phosphate. Xylulose-5-phosphate, a metabolite in the pentose phosphate pathway (PPP), is transformed, along with erythrose-4-phosphate (E4P), into fructose-6-phosphate (F6P) and glyceraldehyde-3-phosphate (G3P) by a transketolase (TKL). Glycerol is probably produced from further metabolization of G3P. X5P metabolization involve E4P metabolization to form F6P and G3P. As E4P is a precursor of erythritol, xylose metabolism is not naturally favorable for erythritol synthesis. This would explain why we did not detect erythritol during growth on xylose.

### The necessity of glucose for erythritol synthesis

When using glucose as carbon source, glycerol, mannitol, and erythritol were measured. Glycerol and mannitol are by-products of glycolysis. Mannitol can originate from F6P, and glycerol can be produced from DHAP. In the presence of a high glucose concentration, we can hypothesize here also that the cell ATP level would be high. This will lead to repression of the PFK, leading to the formation of mannitol, as previously described. It would also lead to the accumulation of G3P, which would lead to the formation of DHAP and the formation of glycerol. The accumulation of F6P and G3P would push the equilibrium of the TKL reaction towards consumption of F6P and G3P and the formation of E4P and X5P. Whereas in the presence of xylose, the equilibrium of the reaction is pushed toward X5P consumption due to high concentration of X5P. This could explain the formation of erythritol only with glucose but not with xylose. An increase of E4P, and thus erythritol, can also hypothetically occur through increased activity of the PPP due to increased glycolysis. An accumulation of glucose-6-phosphate (G6P) would lead to an inhibition of the hexokinase and stimulate the conversion of G6P through the oxidative PPP, leading to the production of E4P and then erythritol. Commonly it is thought that erythritol is formed due to activation of the PPP [9, 27, 40]. However, xylose is considered a PPP activator and did not lead to higher erythritol levels [41, 42]. YE and urea are considered neutral nitrogen sources towards the PPP, but did yield more erythritol than the use of nitrate and ammonium, known to favor the expression of PPP-related enzymes [42–45]. Nitrate is reported to activate the TKL activity, but the direction of the activation is not known [42–45]. Anyhow, the activation of the PPP favors E4P formation through stimulation of the TKL and TAL mediated reactions. However, PPP activation may also lead to E4P consumption through activation of the TKL and TAL reaction towards E4P consumption, emphasizing the complexity of the regulatory network of the PPP with bidirectional reactions.

Our observations suggest that erythritol formation may be tied to glycolysis activation. We would hypothesize that erythritol is formed following high glycolytic activity, leading to the accumulation of glycolysis metabolites and, therefore, stimulating the formation of E4P through the TKL connection to the glycolysis rather than in response to increased flow in the PPP. This hypothesis suggests that erythritol could be used as a marker of heightened glycolytic activity and glucose metabolism overflow. This hypothesis also emerged from research in humans. In human cancer cells A549, erythritol synthesis was reported to be linked to the available glucose concentration and to be a response of TKL activity rather than the G6PDH activity [40]. In addition, plasma erythritol level in humans was found to be associated with fat gain, risk of diabetes, and cancer [46–48]. This suggests that understanding the metabolic link between erythritol and glucose may have a broader impact than improving erythritol bioproduction processes.

## Conclusions

It could be demonstrated that *T. reesei* has the capability to produce erythritol, and this has the advantageous potential to be used to produce erythritol from lignocellulosic biomass residues. The concentration of erythritol presently achievable is lower than in several existing yeast-based processes. Nevertheless, there is still substantial room for improvement through strategic adjustments, such as separating the growth and production phases and optimizing tuning factors like pH, temperature, and medium components. An intriguing aspect of polyol biosynthesis is unraveling the intricate interplay of various parameters. Most of the tested parameters in this study notably influence the quantity and portion of the detected polyols, underscoring a complex regulation governing polyol production. The observation that erythritol appears to be synthesized rather in response to activation of glycolysis than of the PPP adds a layer of complexity to our understanding. Insights into the mechanisms driving erythritol production in eukaryotes may not only result in improved yields for this sugar substitute but also unveil novel targets for diagnosis and treatment of diseases like cancer or diabetes.

### Supplementary Information

Additional file 1. Results of the DoE on the impact of glucose and urea concentrations. *T. reesei* was cultivated in shake flasks on glucose as the sole carbon source and urea as the nitrogen source with concentrations varying from 40 g/L to 90 g/L and 20 to 100 mM, respectively. Two Doehlert designs were performed, both with 3 levels for glucose concentrations and 5 levels for urea concentrations (20 mM, 40 mM, 60 mM, 80 mM, and 100 mM). The first Doehlert was performed with the glucose concentrations 40 g/L, 60 g/L, and 80 g/L (Additional Fig. 1.A) and the second with the levels 50 g/L, 70 g/L, and 90 g/L. Cultivations were performed in triplicates. Samples were taken every 24 h, and glucose and erythritol concentrations were determined by HPLC in the supernatants. Biomass was determined by dry cell weight measurement at the end of cultivation. The results were used to generate a model illustrated by RCPs erythritol concentration (Additional Fig 1.B).

Additional file 2. Calculation of the C/N ratios.

## Data Availability

This published article and its supplementary information files include all data generated or analyzed during this study.

## Abbreviations

6PGDH: 6-Phosphogluconate dehydrogenase
6PGL: 6-Phosphogluconolactonase
DHAP: Dihydroxyacetone phosphate
DK: Dihydroxyacetone kinase
DoE: Design of experiments
E4P: Erythrose-4-phosphate
EP: Erythrose-4-phosphate kinase
ER: Erythrose reductase
F6P: Fructose-6-phosphate
FBA: Fructose bisphosphate aldolase
FDA: Food and Drug Administration
FK: Fructokinase
G3P: Glyceraldehyde-3-phosphate
G3PP: Glycerol-3-phosphate phosphatase
G6P: Glucose-6-phosphate
G6PDH: Glucose-6-phosphate dehydrogenase
GK: Glycerol kinase
GLD: Glycerol dehydrogenase
GPDH: Glycerol phosphate dehydrogenase
GPI: Phosphoglucoisomerase
GRAS: Generally Recognized As Safe
HK: Hexokinase
HPLC: High-pressure liquid chromatography
MDH: Mannitol dehydrogenase
MEX: Malt extract
MPD: Mannitol phosphate dehydrogenase
MPP: Mannitol phosphate phosphatase
PFK: Phosphofructokinase
PPP: Pentose phosphate pathway
RPE: Ribulose-5-phosphate-3-epimerase
RPI: Ribose-5-phosphate isomerase
TAL: Transaldolase
TKL: Transketolase
TPI: Triose phosphate isomerase
XDH: Xylitol dehydrogenase
XKS: Xylulose kinase
XR: Xylose reductase

## References

[1] Rice T, Zannini E, Arendt EK, Coffey A (2020). A review of polyols - biotechnological production, food applications, regulation, labeling and health effects. Crit Rev Food Sci Nutr.

[2] Kunduru KR, Hogerat R, Ghosal K, Shaheen-Mualim M, Farah S (2023). Renewable polyol-based biodegradable polyesters as greener plastics for industrial applications. Chem Eng J.

[3] Uses - European Association of Polyol Producers. https://polyols-eu.org/uses/. Accessed May 2 2024.

[4] Martău GA, Coman V, Vodnar DC (2020). Recent advances in the biotechnological production of erythritol and mannitol. Crit Rev Biotechnol.

[5] Regnat K, Mach RL, Mach-Aigner AR (2018). Erythritol as sweetener-wherefrom and whereto?. Appl Microbiol Biotechnol.

[6] Embuscado ME, Spillane WJ (2006). Chapter 8: polyols. Optimising sweet taste in foods.

[7] Liang P, Cao M, Li J, Wang Q, Dai Z (2023). Expanding sugar alcohol industry: microbial production of sugar alcohols and associated chemocatalytic derivatives. Biotechnol Adv.

[8] Khatape AB, Dastager SG, Rangaswamy V (2022). An overview of erythritol production by yeast strains. FEMS Microbiol Lett.

[9] Rzechonek DA, Dobrowolski A, Rymowicz W, Mirończuk AM (2018). Recent advances in biological production of erythritol. Crit Rev Biotechnol.

[10] Daza-Serna L, Serna-Loaiza S, Masi A, Mach RL, Mach-Aigner AR, Friedl A (2021). From the culture broth to the erythritol crystals: an opportunity for circular economy. Appl Microbiol Biotechnol.

[11] Jeffries TW, Jin YS (2004). Metabolic engineering for improved fermentation of pentoses by yeasts. Appl Microbiol Biotechnol.

[12] Directive - 2009/41/EC. http://data.europa.eu/eli/dir/2009/41/oj. Accessed 4 May 2024.

[13] EFSA Panel on Genetically Modified Organisms (GMO) (2011). Scientific Opinion on Guidance on the risk assessment of genetically modified microorganisms and their products intended for food and feed use. EFSA J.

[14] Vázquez-Vuelvas O, Cervantes-Chávez JA, Delgado-Virgen FJ, Valdez-Velázquez LL, Osuna-Cisneros RJ, De Mandal S, Passari AK (2021). Chapter 7: fungal bioprocessing of lignocellulosic materials for biorefinery. Recent advancement in microbial technology.

[15] Sánchez C (2009). Lignocellulosic residues: biodegradation and bioconversion by fungi. Biotechnol Adv.

[16] de Passos DF, Pereira N, de Castro AM (2018). A comparative review of recent advances in cellulases production by Aspergillus, Penicillium and Trichoderma strains and their use for lignocellulose deconstruction. Curr Opin Green Sustain Chem..

[17] Wang MY, Hou J. Biorefinery of Lignocellulosics for Biofuels and Biochemicals. Quality Living through Chemurgy and Green Chemistry. 2016;143–91. 10.1016/j.cogsc.2018.06.003.

[18] Bischof RH, Ramoni J, Seiboth B (2016). Cellulases and beyond: the first 70 years of the enzyme producer *Trichoderma reesei*. Microb Cell Fact.

[19] Fischer AJ, Maiyuran S, Yaver DS (2021). Industrial relevance of *Trichoderma reesei* as an enzyme producer. Methods Mol Biol.

[20] Meyer V, Basenko EY, Benz JP, Braus GH, Caddick MX, Csukai M (2020). Growing a circular economy with fungal biotechnology: a white paper. Fungal Biol Biotechnol..

[21] Jovanović B, Mach RL, Mach-Aigner AR (2014). Erythritol production on wheat straw using *Trichoderma reesei*. AMB Express.

[22] Gryshyna A, Kautto L, Peterson R, Nevalainen H, Schmoll M, Dattenböck C (2016). On the safety of filamentous fungi with special emphasis on *Trichoderma reesei* and products made by recombinant means. Gene expression systems in fungi: advancements and applications fungal biology.

[23] Frisvad JC, Møller LLH, Larsen TO, Kumar R, Arnau J (2018). Safety of the fungal workhorses of industrial biotechnology: update on the mycotoxin and secondary metabolite potential of *Aspergillus niger*, *Aspergillus oryzae*, and *Trichoderma reesei*. Appl Microbiol Biotechnol.

[24] Metz B, de Vries RP, Polak S, Seidl V, Seiboth B (2009). The Hypocrea jecorina (syn. *Trichoderma reesei*) lxr1 gene encodes a D-mannitol dehydrogenase and is not involved in L-arabinose catabolism. FEBS Lett.

[25] Dashtban M, Kepka G, Seiboth B, Qin W, Dashtban M, Kepka G (2013). Xylitol production by genetically engineered *Trichoderma reesei* strains using barley straw as feedstock. Appl Biochem Biotechnol.

[26] Jovanović B, Mach-Aigner AR, Martzy R (2021). Batch cultivation of *Trichoderma reesei*. *Trichoderma reesei* methods in molecular biology.

[27] Carly F, Fickers P (2018). Erythritol production by yeasts: a snapshot of current knowledge. Yeast.

[28] Sar T, Harirchi S, Ramezani M, Bulkan G, Akbas MY, Pandey A (2022). Potential utilization of dairy industries by-products and wastes through microbial processes: a critical review. Sci Total Environ.

[29] Knežević K, Daza-Serna L, Mach-Aigner AR, Mach RL, Friedl A, Krampe J (2023). Investigation of ion-exchange membranes and erythritol concentration for the desalination of erythritol culture broth by electrodialysis. Chem Eng Process Process Intensif..

[30] Moon HJ, Jeya M, Kim IW, Lee JK (2010). Biotechnological production of erythritol and its applications. Appl Microbiol Biotechnol.

[31] Lin SJ, Wen CY, Liau JC, Chu WS (2001). Screening and production of erythritol by newly isolated osmophilic yeast-like fungi. Process Biochem.

[32] Carly F, Vandermies M, Telek S, Steels S, Thomas S, Nicaud JM (2017). Enhancing erythritol productivity in *Yarrowia lipolytica* using metabolic engineering. Metab Eng.

[33] Carly F, Steels S, Telek S, Vandermies M, Nicaud JM, Fickers P (2018). Identification and characterization of EYD1, encoding an erythritol dehydrogenase in *Yarrowia lipolytica* and its application to bioconvert erythritol into erythrulose. Bioresour Technol.

[34] Rymowicz W, Rywińska A, Marcinkiewicz M (2009). High-yield production of erythritol from raw glycerol in fed-batch cultures of *Yarrowia lipolytica*. Biotechnol Lett.

[35] Guo J, Li J, Chen Y, Guo X, Xiao D (2016). Improving erythritol production of *Aureobasidium pullulans* from xylose by mutagenesis and medium optimization. Appl Biochem Biotechnol.

[36] Yu JH, Lee DH, Oh YJ, Han KC, Ryu YW, Seo JH (2006). Selective utilization of fructose to glucose by *Candida magnoliae*, an erythritol producer. Appl Biochem Biotechnol.

[37] Soerjawinata W, Kockler I, Wommer L, Frank R, Schüffler A, Schirmeister T (2022). Novel bioreactor internals for the cultivation of spore-forming fungi in pellet form. Eng Life Sci.

[38] Jordan P, Choe JY, Boles E, Oreb M (2016). Hxt13, Hxt15, Hxt16 and Hxt17 from *Saccharomyces cerevisiae* represent a novel type of polyol transporters. Sci Rep.

[39] Geddes BA, Pickering BS, Poysti NJ, Collins H, Yudistira H, Oresnik IJ (2010). A locus necessary for the transport and catabolism of erythritol in *Sinorhizobium meliloti*. Microbiology.

[40] Ortiz SR, Heinz A, Hiller K, Field MS (2022). Erythritol synthesis is elevated in response to oxidative stress and regulated by the non-oxidative pentose phosphate pathway in A549 cells. Front Nutr.

[41] Battaglia E, Zhou M, de Vries RP (2014). The transcriptional activators AraR and XlnR from *Aspergillus niger* regulate expression of pentose catabolic and pentose phosphate pathway genes. Res Microbiol.

[42] Masi A, Mach RL, Mach-Aigner AR (2021). The pentose phosphate pathway in industrially relevant fungi: crucial insights for bioprocessing. Appl Microbiol Biotechnol.

[43] Hankinson O, Cove DJ (1972). Regulation of pentose phosphate pathway in fungus *Aspergillus nidulans*. Biochem J.

[44] Hankinson O (1974). Mutants of the pentose phosphate pathway in *Aspergillus nidulans*. J Bacteriol.

[45] Osmond CB, Ap RT (1969). Control of the pentose-phosphate pathway in yeast. Biochim Biophys Acta.

[46] Witkowski M, Nemet I, Alamri H, Wilcox J, Gupta N, Nimer N (2023). The artificial sweetener erythritol and cardiovascular event risk. Nat Med.

[47] Hootman KC, Trezzi JP, Kraemer L, Burwell LS, Dong X, Guertin KA (2017). Erythritol is a pentose-phosphate pathway metabolite and associated with adiposity gain in young adults. Proc Natl Acad Sci USA.

[48] Rebholz CM, Yu B, Zheng Z, Chang P, Tin A, Köttgen A (2018). Serum metabolomic profile of incident diabetes. Diabetologia.

