# Supplementary figures and images for: Exploration of Trichoderma reesei as an alternative host for erythritol production
Source: Biotechnol Biofuels Bioprod. 2024 Jun 27;17:90. doi: 10.1186/s13068-024-02537-x (PMC11210129; doi:10.1186/s13068-024-02537-x)

**Additionnal Fig. 1. A**

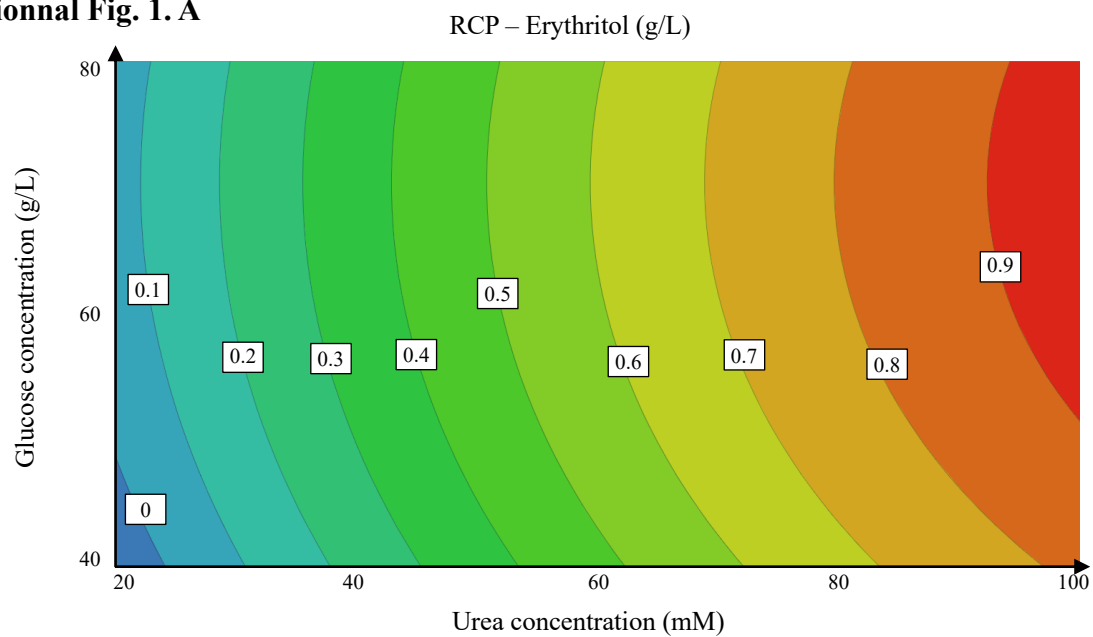

**Additionnal Fig. 1. B**

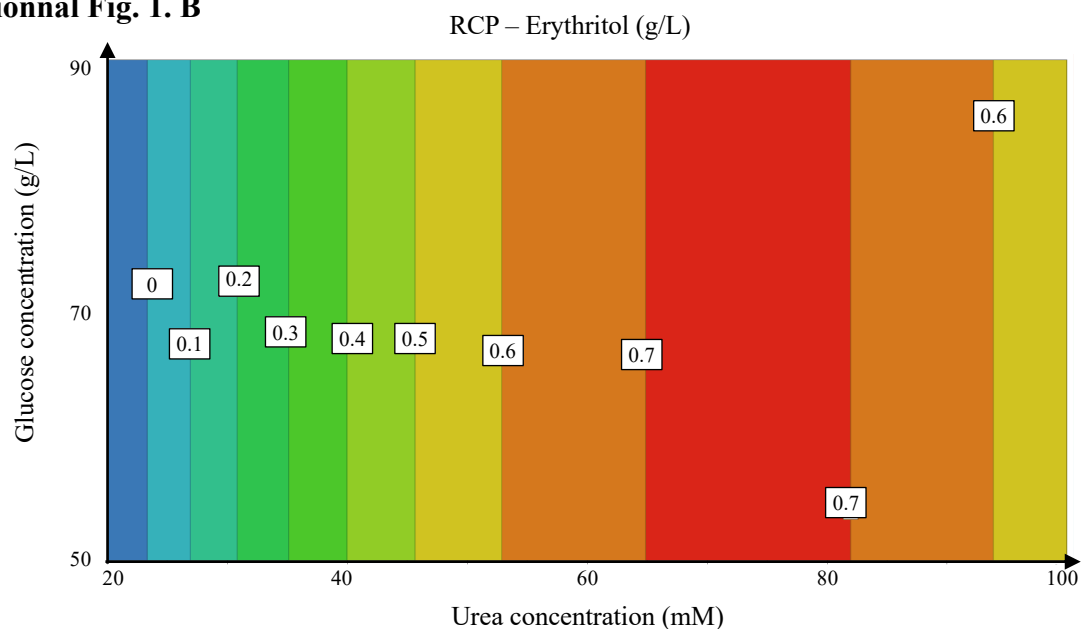

Supplement: Supplementary file 1 — Additional file 1. Results of the DoE on the impact of glucose and urea concentrations. T. reesei was cultivated in shake flasks on glucose as the sole carbon source and urea as the nitrogen source with concentrations varying from 40 g/L to 90 g/L and 20 to 100 mM, respectively. Two Doehlert designs were performed, both with 3 levels for glucose concentrations and 5 levels for urea concentrations (20 mM, 40 mM, 60 mM, 80 mM, and 100 mM). The first Doehlert was performed with the glucose concentrations 40 g/L, 60 g/L, and 80 g/L (Additional Fig. 1.A) and the second with the levels 50 g/L, 70 g/L, and 90 g/L. Cultivations were performed in triplicates. Samples were taken every 24 h, and glucose and erythritol concentrations were determined by HPLC in the supernatants. Biomass was determined by dry cell weight measurement at the end of cultivation. The results were used to generate a model illustrated by RCPs erythritol concentration (Additional Fig 1.B). [file 13068_2024_2537_MOESM1_ESM.pdf]
