# Supplementary material for: Exploration of Trichoderma reesei as an alternative host for erythritol production
Source: Biotechnol Biofuels Bioprod. 2024 Jun 27;17:90. doi: 10.1186/s13068-024-02537-x (PMC11210129; doi:10.1186/s13068-024-02537-x)
Supplement: Supplementary file 2 — Additional file 2. Calculation of the C/N ratios. [file 13068_2024_2537_MOESM2_ESM.docx]

| **Figure** | **Carbon source concentration** | **Carbon moles** | **Nitrogen source concentration** | **Nitrogen moles** | **C/N**  **ratio** |
| --- | --- | --- | --- | --- | --- |
| 2 | Glucose 10 g/L | 0.333 | 20 mM ammonium | 0.02 | 16.6 |
| 2 | Glucose 50 g/L | 1.667 | 20 mM ammonium | 0.02 | 83.1 |
| 2 | Glucose 100 g/L | 3.334 | 20 mM ammonium | 0.02 | 166.3 |
| 3 | Glucose 55.5 mM | 0.333 | 20 mM ammonium | 0.02 | 16.6 |
| 3 | Glucose 277.5 mM | 1.665 | 20 mM ammonium | 0.02 | 83.2 |
| 3 | Xylose 55.5 mM | 0.116 | 20 mM ammonium | 0.02 | 5.8 |
| 3 | Xylose 277.5 mM | 0.974 | 20 mM ammonium | 0.02 | 48.7 |
| 3 | Lactose 55.5 mM | 0.139 | 20 mM ammonium | 0.02 | 6.9 |
| 3 | Lactose 277.5 mM | 1.110 | 20 mM ammonium | 0.02 | 55.5 |
| 4 | Glucose 277.5 mM | 1.665 | 20 mM ammonium | 0.02 | 83.2 |
| 4 | Glucose 277.5 mM | 1.665 | 80 mM ammonium | 0.08 | 20.8 |
| 4 | Glucose 277.5 mM | 1.665 | 20 mM nitrate | 0.02 | 83.2 |
| 4 | Glucose 277.5 mM | 1.665 | 80 mM nitrate | 0.08 | 20.8 |
| 4 | Glucose 277.5 mM | 1.665 | Yeast extract 2 g/L | 0.014 | 118.9 |
| 4 | Glucose 277.5 mM | 1.665 | Yeast extract 8 g/L | 0.057 | 29.2 |
| 4 | Glucose 277.5 mM | 1.665 | 20 mM Urea | 0.04 | 41.6 |
| 4 | Glucose 277.5 mM | 1.665 | 80 mM Urea | 0.16 | 10.4 |
| 5 | Glucose 70 g/L | 2.334 | 80 mM Urea | 0.16 | 14.6 |
